# Can Lp(a) become the next A1C? A case for digital health management tools to overcome inertia to Lipoprotein (a) testing

**DOI:** 10.3389/fcvm.2025.1545787

**Published:** 2025-02-10

**Authors:** Christof Wedemeyer, Martin Peters, Graham Jones

**Affiliations:** ^1^ Patient Solutions & Services, Novartis Pharma AG, Basel, Switzerland; ^2^ Data Design & Clinical Innovation, Novartis Pharmaceuticals, Cambridge, MA, United States; ^3^ Clinical and Translational Science Institute, Tufts University Medical Center, Boston, MA, United States

**Keywords:** awareness, digital support, patient awareness, motivational tools, lipoprotein a, prevention

## Abstract

Despite its known correlations with risk of cardiovascular disease, awareness and testing for Lipoprotein (a) lags that of other serological markers with estimates that less than 1% of the US population have undergone screening. Herein we outline how digital tools framed around motivational models (COM-B and SEM), might help increase likelihood of patients seeking Lp(a) testing as part of their managed care. Furthermore, we highlight how recent trends in prescription of GLP-1 receptor antagonists are serving to motivate patients to manage biomarkers related to T2D and obesity, which are also relevant in cardiovascular disease. Capitalizing on this trend to stimulate interest in Lp(a) management could have near term consequence, and with disease modifying therapies in development ultimately improve outcomes in cardiovascular disease.

## Introduction

Lipoprotein (a) commonly referred to as Lp(a) is a low density lipoprotein that has been linked to increased risk of atherosclerotic cardiovascular diseases which include coronary disease and ischemic stroke (1). Its role in these processes is reasoned to involve concentration dependent buildup on the walls of blood vessels, resulting in plaques which decrease blood flow to major organs and increase the potential for clotting events, aortic stenosis and inflammation. Lp(a) levels are genetically predetermined and although inhibition of apo(a) in liver can reduce Lp(a) in blood, lifestyle modifications such as diet and exercise do not appear to impact Lp(a) levels.

Despite general appreciation of the role of Lp(a) in the cardiovascular medicine community, inertia to testing for this key biomarker prevails despite estimates that some 60 million individuals in the USA live with elevated levels (2), and globally between 20% and 25% of the population is at risk (3). There are also marked disparities among populations with elevated levels in black, female and younger patients and considerable regional variations (4). Compounding this “silent killer burden” the lack of identification of subjects contributes to the barriers faced for clinical trials of investigational agents, which benefit from large patient numbers and high levels of diversity. Exacerbating the problem, historical barriers to clinician mandates for Lp(a) screening exist include uneven reimbursement coverage, exclusion from standard lipid panels offered by laboratories, and lack of availability of Lp(a) lowering therapies (2). The latter situation is now changing rapidly with numerous promising approaches in late stage development (5). Additionally, guidelines for lipid analysis are now signaling the need for Lp(a) screening in certain populations which may help shift momentum (6), and partnerships with consumer based genetic testing services are seeking to further raise awareness (7) given that current estimates suggest that *only 1% of the US population have undergone Lp(a) screening*. Even among patients diagnosed with atherosclerotic cardiovascular disease (ASCVD) <15% have typically had Lp(a) measurements performed suggesting that concerted awareness campaigns are needed to address what can be regarded as a chronic screening gap (4).

Of significance it has been shown that initiation of lipid-reducing therapies was higher for patients who underwent Lp(a) screening in comparison to the more widely adopted LDL-C testing (1, 8), underscoring the benefits of patient engagement. Furthermore, the advent and widespread use of new Glucagon-Like Peptide-1 receptor agonists (GLP-1 RA's) to induce weight loss among T2D and obese patients is having a marked impact on cardiovascular disease awareness, with cardiovascular, renal and metabolic benefits reported among subjects (9, 10). One preliminary study reported that GLP-1 RA's were able to reduce concentrations of Lp(a) by >10% along with several other biomarkers implicated in plaque vulnerability, and these benefits while modest will likely enhance awareness (11). A further bell weather for change is occurring in the wellness industry. Themes such as lifespan, health span and prevention are receiving increased prominence in the popular media and the notion of empowering patients to proactively manage their health status at an early stage is gaining traction (12). The cardiovascular space is course a prime venue for such paradigm shifting approaches to medicine and it would be prudent to develop these themes to address the dearth of Lp(a) screening (13). Given the advent of new therapeutics, improved insight into the psychology of patient motivators, and a rapidly growing understanding of how digital technologies can impact behaviors, we herein outline how smartphone based adjuvants could capitalize on momentum to drive higher screening rates for Lp(a).

## Digital health technologies

The rapid growth in consumer uptake of digital health management tools has been noteworthy, leading to expected lags in adoption by the medical community, as validation and verification requires stringent assessment processes (14). Nonetheless, it is generally appreciated that by raising human awareness to factors which can promote health benefits (e.g., heart rate monitors, sleep assessment devices and activity trackers) positive actions and behaviors can be encouraged (15). Indeed several health coaching and motivational instruments have been developed around digital tools and resulted in marked benefit for patients suffering from cardiovascular disease, T2D and depressive disorders (16). These approaches have demonstrated effectively that human motivation plays a critical role in healthy behaviors and psychological factors are highly influential. Such may be especially important in cardiovascular disease management as its asymptomatic aspects may contribute to historically low medication adherence rates often observed e.g., with statins to reduce LDL-C levels (17).

**Figure 1:**
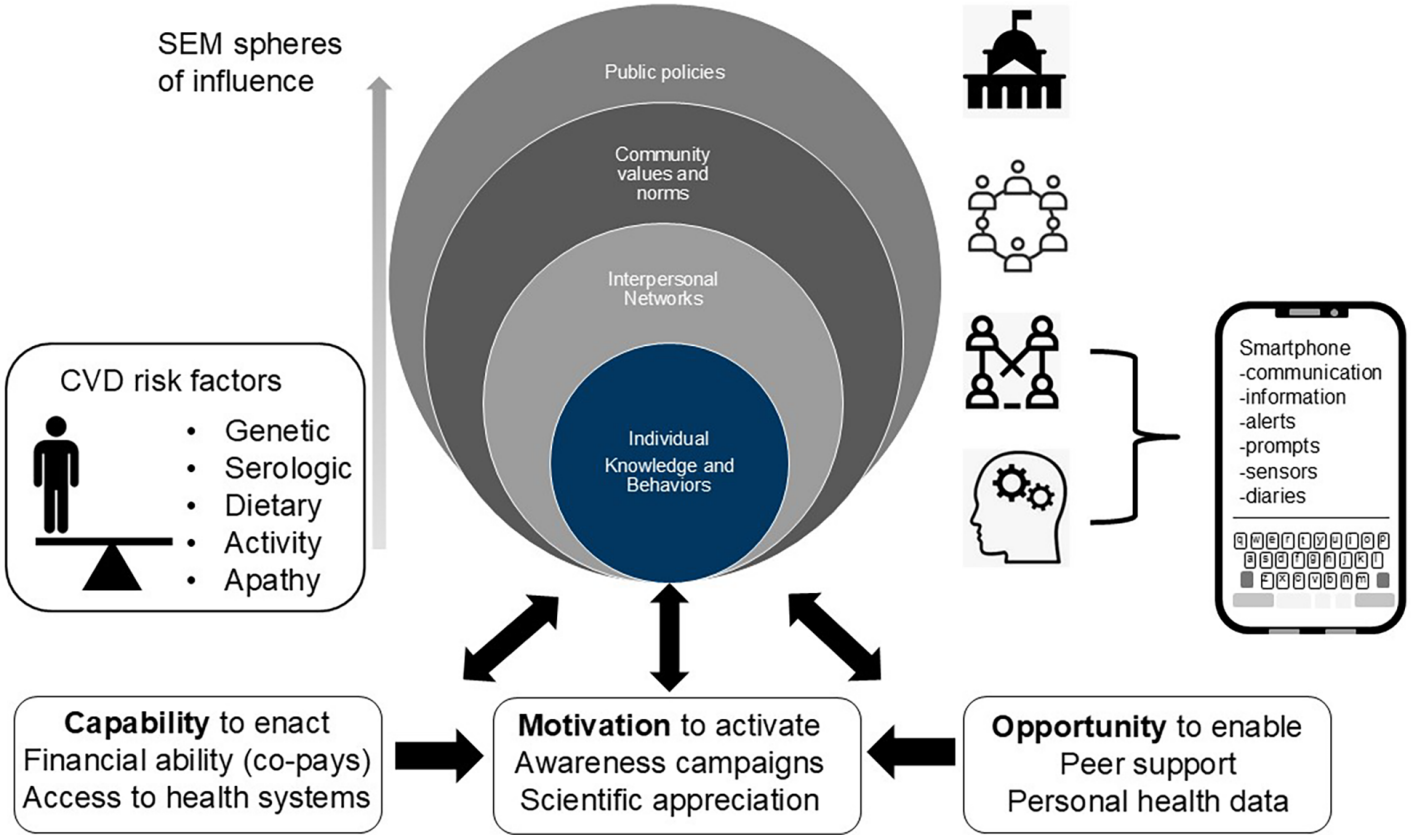
Visualizing factors governing adherent behaviors based on adapted social ecological (SEM, upper) and capability opportunity and motivational (COM-B) models of human behavior (lower) and how smartphone features may influence individual behaviors.

One of the most frequently cited frameworks used in prediction of health behaviors is the COM-B model. Under this approach an individuals actions are reasoned to be governed by the triad of Capability, Motivation and Opportunity and it has been used to analyze medication adherence among myriad applications (Figure 1) (18). In the case of a cardiovascular disease patient, the capabilities to act are influenced by many fixed variables including access to healthcare whereas motivation and opportunity could be amplified by external prompts e.g., from support groups, educational content and awareness campaigns many of which could be delivered through smartphone apps (Figure 1). A related framework is described by Social Ecological Models (SEM) which codify the role of external influences and schemas on individual preferences ranging from public policy through communities and ultimately to individual choices (19). Although these principles were theorized many years ago it is interesting to examine how digital communication tools have influenced how they function in the present day. Given that the smartphone is now a primary means of communication, conducting transactions, performing diary functions, and receiving information from news sources, peers, and health care providers it is reasonable to see its role in deploying the tenets of COM-B and SEM theory for patient benefit.

Obtaining information and medical grade knowledge is a first step towards patient empowerment and can be a springboard for positive behavioral changes. Using the smartphone as a nexus for positive health behaviors is bolstered by the numerous app based functions which can effortlessly capture, store, interpret, and transmit health related data. In the specific area of cardiovascular disease, where the wellness-illness continuum can traverse several decades, designing through this medium would seem to offer obvious advantages in the quest to sustain positive behaviors (13, 17). In cardiovascular disease there are specific defined risk factors to be considered, and must be taken into account in the design of any interventive approach (Figure 1). Genetic predisposition is of course a major determinant that many patients will be oblivious to unless prompted. The advent of rapid, affordable, mail-in genomic testing services coupled with awareness campaigns may address this component, and the results (coupled with appropriate counselling) could stimulate next actions. This could include comprehensive serological evaluation, which should include Lp(a) and other key analytes, and lifestyle modifications which could include dietary modifications, new exercise regimens, and consideration of therapeutic options (Figure 1).

## Designing an app which underscores the importance of Lp(a)

There are in excess of 300,000 health related apps which have been launched over the past decade only a small subset of which are used widely (20). An overwhelming requirement for success is to achieve a careful balance of offering up to date and relevant information for patients and doing so in a manner which enhances experience. This of course will differ from person to person and could involve factors such as audiovisual stimuli, gamification tactics, reward mechanisms or simply ease of use. Of the 3,121 cardiology related apps screened through the mobile app rating scale (MARS), the top two ranked both scored highest prompting *behavioral* change (21), which may relate well to addressing the general apathy associated with preventative measures tied to Lp(a) testing.

While this may hold the key for development of an Lp(a) specific feature on an app, a more compelling and implementable strategy may be to focus on T2D patients who are engaged in active weight management programs including the use of GLP-1 RA's. The International Diabetes Federation (IDF) estimates that there are over 500 million T2D patients globally (approx. 10.5% of the adult population) and it has been reported that up to one third of all T2D patients are living with forms of ASCVD (22). One of the many measures of progress and ultimately success in these cases is lowering of hemoglobin A1C levels to within what is considered normal range (1.0–5.7 g/dl). There is a noted link between A1C and Lp(a) levels in T2D patients and this correlation could be used to prompt patients who are making good tracking progress in lowering of A1C to consider reference testing (23). Such actions represent motivational factors under the COM-B umbrella and could have immediate and widespread impact given the large populations pursuing A1C control (24).

**Figure 2:**
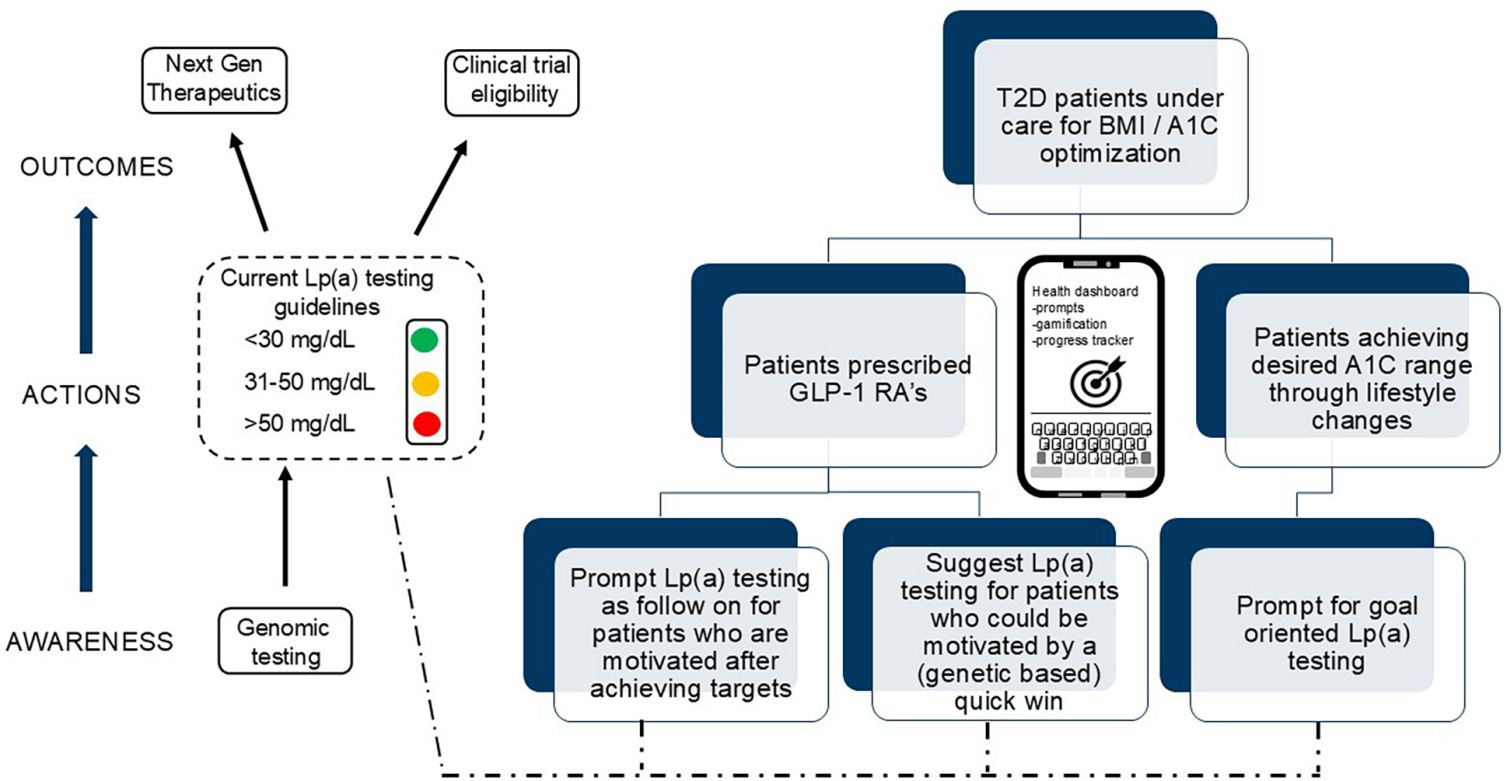
Potential motivating factors and touch points on patient journey linking A1c and genomics to Lp(a).

Such approaches might also help raise awareness among other at risk individuals yet to engage with managed care by encouraging testing. In terms of how such might interface on an app, an EMR dashboard that provides a visual cue on current and target A1C levels could prompt a query on the unknown Lp(a) value, driving curiosity inspired behavioral change (Figure 2). A graphic depicting morbidity and mortality data and known correlations depending on age, sex, BMI and other factors could serve to gamify the approach through a cloaked reference to this “silent killer”. Additional prompts and hooks that reflect on general inertia surrounding Lp(a) testing among HCP’s and the vox populi [e.g., climatology, radon levels, inflation] may be effective means to amplify the message. Alternatively, and resonating with patient phenotypes that are responsive to challenge driven metrics, the need for testing could be framed around an informed end-goal.

What is clear is that several groups of patients including those with T2D, familial history of ASCVD, and populations with elevated risk would be natural candidates for such intervention (4). This could also trigger additional actions, including genomic testing, and entering clinical trials for Lp(a) lowering candidate drugs. There is also the potential to set and frame such an approach around the context of an adverse cardiac event, when awareness and engagement levels would be maximal. Given the centrality of smartphone usage among populations afflicted by T2D (and, within, risk for CVD) it seems logical that a viable system could be developed for field testing among at risk individuals. The benefits of such an approach could be myriad, including helping prompt more widespread genomic testing, increased awareness of interventional therapeutic measures and enrollment in clinical trials (Figure 2).

## Next steps

There is clear need for increased awareness of Lp(a) as a constituent of cardiovascular health as underscored by numerous clinical and epidemiological studies (25), and acknowledgment of the problem featured in popular press articles (26). The evolution of personal digital health technologies and the incorporation of human behavioral factors to drive desired outcomes bodes well for the utilization of app based interventions and adjuvants for at risk individuals pursuing a path to wellness (21, 27). To accomplish the goals outlined herein however will require careful and thoughtful development involving key stakeholders. These include patients and advocate groups, key opinion leaders and experts in cardiovascular health, technology developers, and payers/insurers who can incentivize the use of such tools through managed healthcare. One strategy could be through the deployment of a SMART (Specific, Measurable, Achievable, Relevant, Time-bound) framework. Such could focus on specific goals to e.g., double screening rates within 5 years among the population by awareness campaigns initially focused on those prescribed GLP-1's or under care for ASCVD. This could be augmented with a marketing tactic which engages patients in a similar manner to the ‘crucial catch’ campaign developed by the national football league to raise awareness on prostate cancer screening (28). A logical first step would be for medical professional societies to take a lead and develop a directive which could then be socialized through a third party integrator body who bring together stakeholders. The Critical Path Institute is one such option, and who regularly convene workshops with drug developers, technology developers and policymakers (29). We challenge these constituents to join forces in this endeavor, for the sake of the many millions of patients at risk of major cardiovascular events associated with the silent killer -Lp(a).

## Data Availability

The original contributions presented in the study are included in the article/Supplementary Material, further inquiries can be directed to the corresponding author.
